# Household Air Pollution and Risk of Pulmonary Tuberculosis in HIV-Infected Adults

**DOI:** 10.21203/rs.3.rs-3410503/v1

**Published:** 2023-10-10

**Authors:** Patrick D.M.C. Katoto, Dieudonné Bihehe, Amanda Brand, Raymond Mushi, Aline Kusinza, Brian W. Alwood, Richard N. van Zyl-Smit, Jacques L. Tamuzi, Nadia A. Sam-Agudu, Marcel Yotebieng, John Metcalfe, Grant Theron, Krystal J. Godri Pollitt, Maia Lesosky, Jeroen Vanoirbeek, Kevin Mortimer, Tim Nawrot, Benoit Nemery, Jean B. Nachega

**Affiliations:** Stellenbosch University; Université Evangélique en Afrique; Stellenbosch University; Stellenbosch University; Stellenbosch University; Stellenbosch University; University of Cape Town; Stellenbosch University; University of Maryland School of Medicine; Albert Einstein College of Medicine; Zuckerberg San Francisco General Hospital, University of California; NRF-DST Centre of Excellence for Biomedical Tuberculosis Research, Stellenbosch University; Yale University; University of Cape Town; KU Leuven; Liverpool School of Tropical Medicine; KU Leuven; KU Leuven; Albert Einstein College of Medicine

**Keywords:** Africa, Indoor Pollution, Charcoal, Gender, Health Equity

## Abstract

**Background:**

In developing countries, millions of deaths occur annually from household air pollution (HAP), pulmonary tuberculosis (PTB), and HIV-infection. However, it is unknown whether HAP influences PTB risk among people living with HIV-infection.

**Methods:**

We conducted a case-control study among 1,277 HIV-infected adults in Bukavu, eastern Democratic Republic of Congo (February 2018 – March 2019). Cases had current or recent (<5y) PTB (positive sputum smear or Xpert MTB/RIF), controls had no PTB. Daily and lifetime HAP exposure were assessed by questionnaire and, in a random sub-sample (n=270), by 24-hour measurements of personal carbon monoxide (CO) at home. We used multivariable logistic regression to examine the associations between HAP and PTB.

**Results:**

We recruited 435 cases and 842 controls (median age 41 years, [IQR] 33–50; 76% female). Cases were more likely to be female than male (63% vs 37%). Participants reporting cooking for >3h/day and ≥2 times/day and ≥5 days/weekwere more likely to have PTB (aOR 1·36; 95%CI 1·06–1·75) than those spending less time in the kitchen. Time-weighted average 24h personal CO exposure was related dose-dependently with the likelihood of having PTB, with aOR 4·64 (95%CI 1·1–20·7) for the highest quintile [12·3–76·2 ppm] compared to the lowest quintile [0·1–1·9 ppm].

**Conclusion:**

Time spent cooking and personal CO exposure were independently associated with increased risk of PTB among people living with HIV. Considering the high burden of TB-HIV coinfection in the region, effective interventions are required to decrease HAP exposure caused by cooking with biomass among people living with HIV, especially women.

## Introduction

According to the World Health Organization (WHO), air pollution causes 8·1 million deaths per year, 3·8 million (47%) of which are attributed to household air pollution (HAP) caused by burning biomass for domestic cooking, heating, and lighting.[1] Vulnerable populations at extremes of age, chronically ill, or of low socioeconomic status are at high risk of pollution-related diseases like pneumonia.[2] Approximately 90% of the 10 million new cases of tuberculosis (TB) in 2018 were from low and middle-income countries (LMICs).^3^ In that year, ~ 1·3 million people died from TB, including 251,000 deaths (nearly 20%) among people living with HIV (PLHIV). In 2020, a similar trend was reported, with a substantially greater impact on health services as a result service interruption due to COVID-19 pandemic.[3] Air pollution ranks highest among independent risks in the global burden of disease [4, 5]. HAP has adverse effects on multiple organ systems[6] and has been associated with increased risk of developing TB in the 2020 global burden estimation study.[7] However, we do not know if HAP independently affects TB risk in PLHIV. Based on compositional similarities between tobacco and biomass smoke,[8] it is reasonable to expect that HAP effects on health may be similar to those of tobacco smoke that double the risk of developing TB among PLHIV as compared to their counterpart.[9]

Pulmonary TB (PTB) is associated with tobacco smoking in immunocompromised persons, including PLHIV.[10, 11] Furthermore, lifetime cumulative smoking, rather than daily quantity alone, may be more important in pulmonary outcomes.[12, 13] Similarly, duration of exposure is a major determinant of HAP-associated health consequences. In laboratory simulations, wood-burning three-stone stoves (used for cooking in LMICs) produce approximately 6 g of particulate matter with (aerodynamic diameter ≤ 2·5 μm (PM_2.5_) per hour, equivalent to burning 400 cigarettes. The Democratic Republic of the Congo (DRC) has high burdens of both HIV and TB,[14, 15] and most households utilize solid fuel (wood and charcoal) for cooking.[16] Consequently, HAP interventions might be more cost-effective in this setting than originally thought, as analyses have not previously factored in their potential impact on TB reduction. Little information is available on HAP-related health outcomes in sub-Saharan Africa[17] and, hence, evidence is lacking to inform policy[18].

The WHO guidelines on HAP suggest that PM_2.5_ and carbon monoxide (CO) contribute most to mortality and morbidity caused by incomplete combustion of carbon-based fuels^17^. Although quantifying PM_2.5_ might be ideal for drawing associations between exposure to HAP and health outcomes, PM_2.5_ monitors are cumbersome and expensive, require a stable electrical power supply or batteries, are cumbersome and require shipment and data processing in overseas laboratories. The Lancet Commission on HAP[17] emphasized that CO is simpler to measure than PM_2_·5 and can be used as a proxy for estimating exposure to HAP. Mechanistic studies indicate that CO may by itself exert oxidative stress and immune modulating effects, and epidemiologic studies have shown that CO alone may contribute to the cardiorespiratory effects of air pollution [19–21].

We conducted a case-control study amongst PLHIV to test the hypothesis that PTB risk in this population is associated with sustained exposure to HAP caused by biomass burning in DRC.

## Methods

A detailed description of the methods is available in the electronic Supplement.

### Study design, Population, and Setting

We conducted a case-control study spanning with participants being recruited between March 2018 and February 2019 (Fig. 1) from four major HIV treatment clinics in Bukavu, the capital city of South Kivu province, eastern DRC. All adults ≥ 18 years attending clinic for any reason were approached by clinic nurses; patients interested were then referred to study staff for recruitment. Cases were HIV-positive persons with PTB [(current or within the past 5 years); for each case (HIV+/PTB+), we enrolled two HIV-positive controls without current PTB or history of TB (HIV+/PTB-) who lived in the same neighborhood (same street or village). Given that females in this setting are more likely to cook in most households and be exposed to HAP, we did not perform a matched sampling, to enable us to investigate the effect of gender on PTB risk in stratified analyses[22].

### Variables, Instruments, and Measurements

HIV status was confirmed by HIV registers at recruiting clinics. History of HIV treatment and CD4 cell count (at HIV diagnosis) were extracted from clinical registries as well. Participants were asked about current or history of active TB. PTB history was confirmed by cross-checking against TB registers (laboratory and treatment registries) for those clinics that had ≥ 5 years of records available. Other TB-relevant information such as ZiehlNeelsen stain and/or Xpert MTB/RIF results and TB treatment outcomes were extracted from medical records. In summary, according to the Congolese National Tuberculosis Programme (NTP)[23], PTB was diagnosed based on a consistent clinical history and confirmation with Ziehl-Neelsen sputum smear microscopy for acid-fast bacilli (AFB). Notably, in this high-burden TB situation with limited resources, only a selected number of patients can benefit from Xpert and X-ray examinations due to logistical constraints (data not shown) caused by a decade of armed conflict in the region[24]. Processes were similar for both cases and controls at all recruiting facilities. Hospital-based study staff were not blinded to patient HIV/TB status, since they were responsible for verifying eligibility from patient records. However, field study staff who collected CO data were blinded to status for methodological reasons and to avoid stigma. To reduce the risk of misclassification and (non-differential) bias related to changes in behavior, we used a generic information script.

All questionnaires were administered face-to-face by trained interviewers. A short questionnaire assessed socio-demographic characteristics (age, gender, marital status, educational level, employment, and income), location of residence (urban vs rural), alcohol consumption, and active or passive tobacco smoking.

To assess HAP exposure, we used the International Multidisciplinary Programme to Address Lung Health and TB in Africa (IMPALA) questionnaire [25] to obtain information on housing type (separate kitchen, roof type, ventilation); indoor/outdoor kitchen location; cooking modes [“three stones” (Fig. 2), cookstove, kerosene/electric stove]; and lighting/heating methods. Since > 95% of DRC households utilize biomass fuel for domestic energy,[26] we anticipated little or no differences in the prevalence of such exposure between cases and controls. We defined – before conducting any statistical analyses – proxies of cumulative intensity of exposure based on time spent cooking daily and during adult life. Hence, a composite index of “high exposure” was given to participants who reported spending ≥ 3 hours in the kitchen daily AND cooking at least twice daily AND cooking five or more days a week. To estimate lifetime exposure to cooking, we asked at what age participants had prepared their first “ugali”, a common maize flour-based meal. Preparing ugali is traditionally seen as a sign of maturity and implies that one may take charge in the household’s kitchen. We considered actual age minus age at first ugali to reflect lifetime spent in the kitchen and dichotomized this variable into less or more than 25 years since first ugali. Time spent in the kitchen was then adjusted for age at first ugali, and type of primary fuel was used to generate a proxy for cumulative HAP exposure. Of note, some men also worked as cooks for wealthier families.

Computer-generated random numbers were used to select a sub-sample of 105 non-smoking cases; these cases and 165 of their corresponding non-smoking controls were invited for measurement of personal 24h CO exposure, using portable CO data loggers with a USB interface (Lascar monitor, El-USB-CO^®^). After explaining the purpose of measuring personal CO, a trained physician (PDMK) placed the monitor on participants’ clothing (Fig. 2, panel C), at their home, between 6 and 8 AM, encouraged them to do their normal activities until next day, when the recording was ended and data were downloaded. Time-weighted average (TWA) and maximum/minimum CO concentrations were generated using manufacturer software. The personal CO results were later discussed with each participant, together with giving advice on ventilation, cooking habits, and the benefits of clean energy.

### Sample Size and Power

A study in Benin demonstrated a significant association between HAP and PTB with an estimated odds ratio of 1.7[27]. We, therefore, estimated the number of cases and controls needed to detect a similar effect. Assuming 80% of controls were exposed to HAP, we needed 414 cases and 828 controls (2:1 controls-cases ratio) to demonstrate a significant odds ratio of at least 1.7 with 80% power at 5% significance level. Ultimately, a total sample size of 1,277 participants (435 cases and 842 controls) were recruited. Having two controls to one case increases power over 1:1 controls-cases ratio[28].

### Data Analysis

Categorical data are reported as proportions, and continuous data as means (± standard deviation, SD) or medians (interquartile range, IQR). To check for normality, we used visual inspection of boxplots and the D’Agostino-Pearson omnibus normality test. CO levels were log-transformed. Chi square tests (for categorical variables) and unpaired t-tests or MannWhitney U tests (for continuous variables) tested differences between cases and controls. We used multivariable logistic regression analyses to assess associations between socio-demographic, clinical, and domestic energyrelated predictors, CO levels (continuous and by quintiles) and risk of having PTB. We estimated adjusted associations by including all baseline covariates (including CD4 count and ART duration) *a priori* in a multivariable model. Starting with a full model, we then used a backward elimination procedure, excluding predictor variables with a p-value>0·1, and compared the estimated reduced model adjusted odds ratios (aOR) and associated 95% confidence intervals (CIs), with the full multivariable model estimates. The final model was based on variables with statistical significance in both the full and reduced models, plus variables with possible clinical significance. Since women are more likely to be exposed to HAP than men[17, 29], we conducted a stratified analysis by gender to remove gender-related confounding. All p values were two-sided, and p< 0·05 was considered statistically significant. Analyses were performed using Stata/SE 14 (Stata Corp, College Station, TX, USA) and GraphPad Prism 8.1.1 (GraphPad Software Inc., San Diego, CA, USA).

### Research Ethics Approval

#### Ethical approval

was obtained from the Institutional Review Board of the Catholic University of Bukavu (UCB/CIE/NC/01/2018).

## Results

### Socio-Demographic and Household Air Pollution Exposure Data

We included 1,277 participants: 435 cases (HIV+/TB+) and 842 controls (HIV+/TB−). Median (IQR) age was 41 (33–50) years, and 76% were female (Table 1). Half of participants had only a primary education, and half were housekeepers or unemployed. There were more current/former smokers among cases (13%) compared to controls (9%). Similarly, second-hand smoke exposure was more frequent among cases (31 %) than controls (22%). Cases were more likely to be female than male (63% vs 37%). Among the 435 cases, 197 (45%) were currently receiving TB treatment, and 238 (55%) had been diagnosed and treated for TB within the past 5 years. Median [IQR] CD4 count (cells/μL) at HIV diagnosis was lower among cases (219 [110–381]) than controls (282 [151–484]) (Table 1).

Roughly twothirds of kitchens were located inside homes, with only half having a chimney (Table 2). Exclusive use of biomass fuel for cooking was higher among cases (34%) than controls (29%) (p = 0.07). According to our composite index, a significantly higher proportion of cases (47%) had high exposure in the kitchen than controls (40%). Additionally, among cases, 74% had spent > 25 years cooking since first ugali, versus 68% among controls.

Valid CO measurements were obtained for 255 of the 270 randomly selected participants: 96 cases [70 (73%) women] and 159 controls [127 (80%)] women]. Maximum and TWA values [median (IQR) ppm] of 24h personal CO concentrations did not differ significantly between cases [101 (52–223) and 6 (2–12), respectively] and controls [91 (47–155) and 5 (2–9), respectively; eFigure 1 and eTable 1], neither did they differ when stratified by sex (eFigure 2).

### Associations Between Sociodemographic Data, Clinical Profiles, and Risk of Pulmonary TB

After holding other variables constant (including CD4 count and ART duration) in the model, women appeared protected against TB risk (aOR 0·39; 95%CI: 0·27 – 0·55) (Table 3). Furthermore, CD4 count < 200 cells/μL as well as unknown CD4 count were independently associated with TB, with aORs of 2·05 (1·28 – 3·31) and 1·69 (1·06 – 2·70), respectively.

### Associations Between Exposure to HAP and Risk of Pulmonary TB

Overall, after adjusting for known confounding factors (including CD4 count and ART duration), participants with “high exposure” in the kitchen were 1·36 (95% CI: 1·06 – 1·75) times more likely to have TB than those spending less time cooking (Table 4). Among cooks with < 25 years since first ugali, exclusive use of “three stones” for cooking increased TB risk fourfold (aOR 4·05; 95% CI: 1·83 – 8·96). Within this group of younger cooks, cooking indoors, which implies using alternate cooking energy sources, was independently associated with a halving of TB risk (aOR 0·46; 95% CI: 0·26 – 0·81).

The 24-hour TWA of personal CO was positively associated with the odds of pulmonary TB (aOR 1·50 for a log_10_ increase in TWA CO; 95% CI: 1·01–2·23) (Table 5), with an even higher estimate when only women were considered (aOR 4.24; 95% CI 1.09–16.49). In a model with quintiles of 24h personal TWA CO, and adjustments for socio-demographic, clinical, and household energyrelated predictors, we found an exposure-dependent increase in odds of TB from the lowest quintile [0·1–1·9 ppm] to the highest quintile [12·3–76·2 ppm] (Fig. 3 and eTable 2).

## Discussion

Our case-control study provides evidence for a robust association between high HAP exposure from biomass smoke and risk of TB among adult PLHIV. Using a validated questionnaire, we found that high daily and cumulative exposure to HAP increased TB risk. Based on domestic CO measurements, we showed an exposure-response relationship between CO concentration and PTB risk, with participants in the highest quintile of 24h personal CO TWA concentrations exhibiting a fourfold higher risk of having developed TB than those in the lowest quintile, independently of other variables.

We found that “time spent in the kitchen” was a predictor of PTB, particularly for women, despite the well-known, protective role of female gender against TB [30–32]. When stratified by “years since first ugali”, the variable “time spent in the kitchen” remained independently associated with PTB among participants in the highest stratum (> 25 years cooking ugali). For younger participants with < 25 years since first ugali, using only the traditional “three stones” for kitchen activities increased PTB risk. However, among these younger cooks, cooking inside the main house (a proxy for using a non-biomass energy source) was associated with a reduced PTB risk, especially if a chimney was present. In Africa, people in urban or suburban areas typically have two kitchens – an indoor one using electricity or gas, and an outdoor one using solid fuel. However, in the DRC, ownership of electrical appliances (56% in our study) is not a good indicator for low HAP exposure, because an inconsistent power supply forces many to rely for much of the time on non-electricity sources, such as biofuel[33]

Our findings from the questionnaire were largely corroborated by personal CO measurements, which were obtained for 20% of participants. In this subgroup, TB risk was significantly associated with “time spent in the kitchen”. In addition, there was a significant and dose-dependent association with TWA 24h personal CO, but not with maximum 24h personal CO levels. The lack of association between maximum CO and PTB suggests that prolonged exposure is needed to affect the risk of PTB.

HAP and tobacco smoke similarly affect human health. Meta-analyses have shown that tobacco smokers are about twice as likely to develop (fatal) PTB than non-smokers.[34–36] Over the last decade, conflicting data have been published about the relationship between HAP exposure from biomass and PTB risk.[17, 37] Two systematic reviews found very low-quality evidence for an additional risk of TB in relation to HAP exposure. [38, 39] Conversely, an association between solid fuel use and TB was found in an analysis of thirteen studies conducted between 1996 and 2012.[40] Ten of these studies yielded a pooled OR of 1·30 (95% CI 1·04–1·62), while six yielded a pooled OR of 1·70 (1·10–8·20) in a subgroup analysis considering gender. A meta-analysis[41] including twelve studies reported an overall effect estimate of 1·43 (95% CI 1.07–1·91) and, among women, 1·61 (95% CI 0·73–3·57). Finally, a 2020 global systematic review (53 studies) estimated a pooled relative risk of 1·26 (95%CI 1·08 – 1·48) of PTB associated with HAP exposure^6^. This association has been less studied among PLHIV, who are the most vulnerable to TB.[2, 6, 17] The lungs have been described as an anatomic reservoir of HIV,[42] and tobacco smoking is known to induce pulmonary immune defects.[43] Interactions between TB-HIV-tobacco smoking and non-communicable lung diseases have been demonstrated in several studies and summarized in two reviews by van Zyl-Smit and colleagues.[10, 11]

The pathophysiologic mechanisms causing lung illnesses after HAP exposure are not fully understood and while combustion might produce both CO and PM2.5, their ability to impair cell immunity might be different. The current evidence pertaining to HAP mechanistic effects was summarized by the European Respiratory Society/American Thoracic Society task force on HAP.[8] Based on this expert panel that reviewed both cell culture and animal studies, it appeared that numerous HAP-related health effects are also a consequence of impaired bacterial phagocytosis in alveolar macrophages loaded with carbon.[43, 44] From ambient air pollution studies, we know that human -defensin 2 and 3 expression in M. tuberculosis-infected A549 cells was reduced by exposure to PM2.5 or PM10[45]. The ability of cells to control M.tb growth and the M.tb-induced expression of CD69, an early surface activation marker expressed on CD3 + T cells, as well as the production of IFN-, TNF-, and TBX21 in M.tb-infected PBMC, were all reduced when exposed to PM2.5 prior to M.tb infection[46]. This suggests biological pathways underpinning changed M.tb infection and treatment results when exposed to PM2.5[47].

Time spent in the kitchen proved to be a determinant of risk for TB. In addition to doubling the risk of developing TB among PLHIV, long-term tobacco smoking has been shown to attenuate both immune and antiviral responses to antiretrovirals by as much as 40%.[9] At the cellular level, our findings could partly be explained by recent evidence that cytokine production by alveolar macrophages (AM) is inversely related to chronic biomass smoke exposure.[8] Moreover, the association we observed between CO exposure and PTB might be explained by impaired oxidative responses.[8, 44, 48] Compared to PM, it is know that lung and systemic M. tuberculosis-induced cytokine production are altered by PM load in AM and that chronic PM exposure with elevated proinflammatory cytokine expression leads to cellular inactivity.[49] In addition, the pulmonary compartment contains many macrophage-specific immunological deficiencies in smokers, which may explain how smoking makes a patient prone to TB infection and illness.[50] As a result, cigarette smoke reduces effector cytokine responses and inhibits mycobacterial containment inside infected human macrophages from the peripheral blood and alveolar compartments.[51] After Mtb infection, human AM show metabolic plasticity that facilitates glycolytic reprogramming. Smokers also have reduced metabolic reserve, impairing the glycolytic response to infection.[52] Our results, showing a 1·4-fold increase in the odds of developing PTB after chronic high exposure to biomass smoke, are compatible with these findings. It is also wellestablished that level of impairment is more severe with exposure to wood smoke than to fine carbon black, [8] and we demonstrate here that exclusive wood use for cooking increased the likelihood of having developed TB approximately fourfold among younger cooks with HIV infection. Future mechanistic studies (causal mechanisms between the environment and the host response to tuberculosis) should consider HIV-infection status (level of immune defence vs pollutant dose-response) and evaluate if other pollutants (multipollutant model), such as volatile organic compounds (e.g., benzene metabolites), also interfere with mechanisms affecting PTB (risk, new *Mycobacterium tuberculosis* rate, clinical outcomes) as recently suggested by a study on latent TB infection in Vietnam where PM2.5 did not show a significant association as expected.[53, 54]

During the past decade, major randomized trials in LMICs, such as the RESPIRE (Guatemala)[55], CAPS (Malawi)[56], and currently the GRAPHS (Ghana)[57] trials, have used CO as a surrogate for HAP exposure. Similar to our findings, a large nested case-control, single pollutant study in California (2,309 cases and 4,604 controls)[19] found an association between quintiles of CO and TB risk, whereas no association was found for quintiles of PM_2_.5. However, it is known that CO and particulates do not always correlate.[58] We need simple, affordable, and reliable markers of exposure ^4,8,34^ to inform well-designed interventional studies to reduce HAP-induced chronic lung disease.

As part of worldwide TB control efforts, initiatives to integrate tobacco cessation with air pollution reduction (e.g., supply of inexpensive clean energy sources, identification, monitoring, and reduction of air pollution sources) should be addressed. Such initiatives might involve developing patient- and community-focused air pollution mitigation methods and interventions in collaboration with governmental entities, such as patient-screening tools for air pollution risk in at high-risk patients for TB (eg., PLHIV by targeting woman and patients deeply immunocompromised). Interventions should address technology (e.g., improved solid fuel stoves such as traditional and modern combustion designs using fans or gasification equipment), fuel type (e.g., unprocessed or processed such as pellets for biomass, briquettes for coal as well as cleaner fuels such as liquid petroleum gas, biogas, permanent electricity, solar lights), better ventilation system (e.g., chimneys, opening windows while cooking), and behavioral adjustments (e.g., enables for cleaner technologies and fuels such dryness of fuel and community endorsement by community leaders or religious).[17, 60] In light of the current COVID-19 pandemic that has hampered several efforts to eradicate TB, preventing future TB requires addressing not just the disease but also the major drivers of TB (undernutrition, poverty, diabetes, cigarette use, and household air pollution) if TB is to be eradicated by 2035.[61] Hence, our study has significant implications for addressing the global respiratory health threat in LMICs, which is fuelled by a high prevalence of chronic respiratory diseases (asthma, chronic obstructive pulmonary disease, bronchiectasis, and post-tuberculosis lung disease), COVID-19 infection and long COVID-19, all of which are associated with environmental factors and endemic HIV.[62–65] In Box 1, we have summarized and contextualized the implications of our findings in the fight against tuberculosis. Several further strengths of our study include the following: : 1) A large sample size, which adequately powered for our primary objective to investigate HAP-associated PTB risk in PLHIV; 2) Combination of a validated IMPALA questionnaire and 24h personal CO monitoring data; 3) Quantification of cumulative exposure to HAP using simple indexes adaptable/generalizable to other LMIC settings; 4) Documentation of an exposuredependent relationship between HAP and TB risk; 5) Findings suggesting increased PTB risk for women, as they are more likely to be exposed to HAP in our DRC setting.

Although informative, our study also has several limitations. Questionnaire responses and our proxy for lifetime exposure may have been affected by recall bias; however, it is unlikely that responses were influenced by case or control status, both of which were defined using reliable objective criteria. Besides, such recall bias would tend to dilute the effect size, thus making our findings conservative and hence more compelling. Nevertheless, to mitigate recall bias, trained interviewers used the IMPALA questionnaire, and multiple sources were used to triangulate information. We did not account for unmeasured confounding, such as nutritional status and overcrowding, but included surrogates for socio-economic status, such as educational attainment and income. We also relied on one-time 24h personal CO measurements, which might underestimate or overestimate true effect size. However, recent data have linked short term (three-month) exposure to air pollutants (PM, CO, etc.) and increases in TB incidence.[20] We did not consider meteorological factors, but these are unlikely to have introduced systematic biases as the weather varied little during the study, and CO measurements were consistently taken during the same time-period for cases and controls. As indicated before, we acknowledge that CO may not be the best indicator of HAP exposure from biomass smoke. It would have been desirable to measure fine particulate matter, possibly the most harmful component of biomass smoke,[29] or other biomarkers of wood smoke exposure such as urinary guaiacol or levoglucosan.[8] In the absence of ambient air quality monitoring in South-Kivu – as in most areas in Africa[66] – we did not take outdoor air pollution into account. However, ambient air pollution is unlikely to have differed between cases and controls because both were recruited from the same small geographical area. Future research should explore long-term exposure monitoring and/or a biomarker of HAP exposure. However, a multipollutant model that measures both PM2.5 and CO utilizing a low-cost, long-lasting battery sensor may be beneficial for orienting intervention in environments with variable resources. Finally, there was unequal gender distribution,[22] due to the high proportion of women among PLHIV, on one hand, and higher risk of having TB among men, on the other. We addressed this issue by performing sex-stratified analyses.

In conclusion, personal CO exposure and time spent cooking (among women) were found to be independently associated with increased odds of PTB among PLHIV. Public health implications of our findings, if confirmed by further longitudinal studies, are that HAP interventions might prove cost-effective if reductions in TB are considered and measured since HAP is modifiable. Furthermore, progress in the fight against TB might be stalled if we do not adequately address HAP, which is an increasing problem in LMICs with high burdens of both HIV and TB.

## Figures and Tables

**Table 1:** Socio-Demographics and Clinical Data Among 1277 HIV-infected Outpatients Attending ART-Clinics in South Kivu, DRC

Variables	All (N = 1277)	Cases (N = 435)	Controls (N = 842)
**Socio-Demographic Variables**			
**Age** (years)*	40·6 [33–50]	41 [34–51]	40 [32–50]
**Gender**			
Male	312 (24·4)	161 (37·0)	151 (17·9)
Female	965 (75·6)	274 (63·0)	691 (82·1)
**Marital Status**			
Married	571 (44·7)	204 (46·9)	367 (43·6)
Separated	150 (11·7)	55 (12·6)	95 (11·3)
Single	556 (43·5)	176 (40·5)	380 (45·1)
**Level of Education**			
University	55 (4·3)	17 (3·9)	38 (4·5)
High School	458 (35·9)	153 (35·2)	305 (36·2)
Primary School	586 (45·9)	217 (49·9)	369 (43·8)
No School	178 (13·9)	48 (11·0)	130 (15·4)
**Occupation**			
Public Function	86 (6·7)	27 (6·2)	59 (7·0)
Farmer	22 (1·7)	10 (2·3)	12 (1·4)
Private sector	221 (17·3)	62 (14·3)	159 (18·9)
Housekeeper	645 (50·5)	235 (54·0)	410 (48·7)
None at all	303 (23·7)	101 (23·2)	202 (24·0)
**Household Members**			
< 5	271 (22·9)	89 (22·4)	182 (23·2)
≥ 5	910 (77·1)	308 (77·6)	602 (76·8)
**House Roofing**			
Sheet metal	1,087 (85·1)	360 (82·8)	727 (86·3)
Tiles	26 (2·0)	11 (2·5)	15 (1·8)
Straw	164 (12·8)	64 (14·7)	100 (11·9)
**House Wall**			
Brick/Stone	346 (27·1)	135 (31·0)	211 (25·1)
Wood/Plank	561 (43·9)	176 (40·5)	385 (45·7)
Mud	355 (27·8)	115 (26·4)	240 (28·5)
Straw	15 (1·2)	9 (2·1)	6 (0·7)
**Alcohol drinking**			
Never			
Ever	821 (66·8)	282 (66·5)	545 (67·0)
Regular	411 (33·2)	142 (33·5)	269 (33·0)
**Tobacco Smoking**			
Never	1,106 (90·6)	365 (86·9)	741 (92·5)
Ever^¥^	115 (9·4)	55 (13·1)	60 (7·49)
**SecondHand Smoke (among non-current smokers)**			
No	872 (75·2)	273 (69·5)	599 (78·2)
Yes	287 (24·8)	120 (30·5)	167 (21·8)
**Clinical Variables**			
CD4 at HIV diagnosis (cells/μL)* ^$^	261 [139–442]	219 [110–381]	282 [151–484]
≥ 500	174 (13·6)	43 (9·9)	131 (15·6)
200–499	350 (27·4)	110 (25·3)	240 (28·5)
< 200	313 (24·5)	119 (27·4)	194 (23)
Unknown	440 (34·5)	163 (37·5)	277 (32·9)
**ART Duration (years)** *	5 [3–9]	5 [3–9]	5 [3–9]
≥ 10	249 (19·5)	90 (20·7)	159 (18·9)
9-May	465 (36·4)	150 (34·5)	315 (37·4)
< 5	373 (29·2)	122 (28·1)	251 (29·8)
Unknown	190 (14·9)	73 (16·8)	117 (13·9)
**Case Contact Household Past 5 Years**			
Yes	472 (40·9)	172 (41·2)	300 (40·7)
No	683 (59·1)	245 (58·8)	438 (59·3)
**ART initiation compared to TB treatment**			
Before TB treatment	NA	86 (29·5)	NA
Likely same Period	NA	90 (30·8)	NA
After TB treatment	NA	116 (39·7)	NA
**Hospitalisation for TB**			
No	NA	207 (49·8)	NA
Once	NA	163 (39·2)	NA
Twice or More	NA	46 (11·1)	NA
**TB Category**			
New case	NA	324 (86·6)	NA
Retreatment	NA	17 (4·5)	NA
Abandoned	NA	30 (8·0)	NA
Failure	NA	3 (0·8)	NA
**DOT at Hospital for 2 Months**			
Yes	NA	272 (66·5)	NA
No	NA	137 (33·5)	NA

Data are numbers (percentages) except for variables with an asterisk * where median and interquartile ranges [IQR] are shown. Numbers do not always add up to indicated totals because of missing answers. P-values refer to difference between cases and controls.

$: at diagnosis is related to at the time of HIV-infection diagnosis.

ART: antiretroviral therapy; DOT: directly observed treatment; MTB/RIF: polynuclear chain reaction to detect resistance against rifampicin; NA: not applicable

¥: current and former smokers during the interview’ period.”

**Table 2:** Domestic Energy and Kitchen Related Exposure Among 1277 HIV-Infected Outpatients Attending ART-Clinics in South Kivu, DRC

Variables	All (N = 1277)	Cases (N = 435)	Controls (N = 842)	P-value
**Kitchen Location**				
Outside main House	400 (31·3)	152 (34·9)	248 (29·5)	0·04
Inside main House	877 (68·7)	283 (65·1)	594 (70·5)	
**Ventilation System**				
Yes	645 (52·2)	233 (54·7)	412 (50·9)	0·2
No	591 (47·8)	193 (45·3)	398 (49·1)	
**Principal Kitchen Instrument**				
Electric/gas cooker	661 (56·4)	214 (52·8)	447 (58·2)	0·08
Cookstove	226 (19·3)	77 (19·0)	149 (19·4)	
Three Stones	286 (24·4)	114 (28·1)	172 (22·4)	
**Only BMF for Cooking**				
No	806 (69·2)	267 (65·9)	539 (70·9)	0·07
Yes	359 (30·8)	138 (34·1)	221 (29·1)	
**Only Wood for Cooking**				
No	1,232 (96·5)	412 (94·7)	820 (97·4)	0·01
Yes	45 (3·5)	23 (5·3)	22 (2·6)	
**Improved Cookstove used > 10 years**				
Yes	684 (55·1)	212 (50·0)	472 (57·8)	0·009
No	557 (44·9)	212 (50·0)	345 (42·2)	
**Heating Mode**				
Charcoal	622 (58·2)	210 (56·3)	412 (59·3)	0·34
Wood	446 (41·8)	163 (43·7)	283 (40·7)	
**Lighting Mode**				
Clean fuel	629 (49·3)	219 (50·3)	410 (48·8)	0·6
Unclean fuel	646 (50·7)	216 (49·7)	430 (51·2)	
**Time with Electricity per 24h**				
≥ 5h	233 (41·6)	73 (43·5)	160 (40·8)	0·56
< 5h	327 (58·4)	95 (56·5)	232 (59·2)	
**Time Spent in Kitchen** ^#^				
Low exposure	744 (58.3)	232 (53·3)	512 (60·8)	0·01
High exposure	533 (41·7)	203 (46·7)	330 (39·2)	
**Years since first Ugali** ^##^				
Continuous (years)*	28 [19–36]	28 [19–38]	28 [19–36]	0·1
Categorical (< 25 years)	385 (30.2)	112 (25.8)	273 (32.4)	0·01
Categorical (≥ 25 years)	892 (69·9)	323 (74·3)	569 (67·6)	

Data are numbers (percentages) except for variable with an asterisk where medians and interquartile ranges (IQR) are shown. Numbers do not always add up to indicated total because of missing answers. P-values refer to difference between cases and controls. BMF: biomass fuel.

# Time spent in kitchen estimated by a composite indicator based on three sub-variables: participants were classified as highly exposed if they scored above the cut-off values for each sub-variable (cooking > 3h/day and ≥ 2 times/day and ≥ 5 days/week).

## Years since first ugali: current age minus age when preparing first ugali (maize-based porridge) for the household, used as proxy for lifetime duration of domestic cooking.

**Table 3:** Multivariable Analysis: Socio-demographic and Clinical Indicators as Predictors for Pulmonary Tuberculosis Among 1277 HIV-Infected Outpatients Attending ART-Clinics in South Kivu, DRC

Variables	Univariate Analysis			Multivariable Analysis #		
	N	cOR (95%CI)	P-value	N = 956	aOR (95%CI)	P-value
**Gender**	1277					
Males		Ref				
Females		0·37 (0·28 – 0·48)	< 0·001		0·39 (0·27 – 0·55)	< 0·001
**Marital Status**	1277					
Married		Ref				
Separated		1·04 (0·71 – 1·51)	0·83	¨	¨	¨
Single		0·83 (0·65 – 1·07)	0·15	¨	¨	¨
**Level of Education**	1277					
University		Ref				
High School		1·12 (0·61 – 2·05)	0·71	¨	¨	¨
Primary School		1·31 (0·72 – 2·38)	0·37	¨	¨	¨
No School		0·82 (0·42 – 1·60)	0·57	¨	¨	¨
**Occupation**	1277					
Public Function		Ref				
Famer		1·82 (0·70 – 4·73)	0·22	¨	¨	¨
Private sector		0·85 (0·50 – 1·46)	0·56	¨	¨	¨
Housekeeper		1·25 (0·77 – 2·03)	0·36	¨	¨	¨
None at all		1·09 (0·65 – 1·83)	0·74	¨	¨	¨
**Household Members**	1,181					
< 5		Ref				
≥ 5		1·05 (0·78 – 1·40)	0·76	¨	¨	¨
**Alcohol**	1238					
No		Ref				
Yes		1·02 (0·80 – 1·31)	0·88	¨	¨	¨
**Tobacco Smoking**	1221					
Never		Ref				
Evers		1·86 (1·26 – 2·26)	0·002		1·08 (0·67 – 1·74)	0·75
**SecondHand Smoke**	1159					
No		Ref				
Yes		1·58 (1·20 – 2·08)	0·001		1·41 (0·64 – 1·50)	0·8
**CD4 at HIV diagnosis (cells/μL)**	1277					
≥ 500		Ref				
200–499		1·40 (0·92 – 2·11)	0·11		1·33 (0·83 – 2·14)	0·23
< 200		1·87 (1·24 – 2·82)	0·003		2·05 (1·28 – 3·31)	0·003
Unknown		1·79 (1·21 – 2·66)	0·004		1·69 (1·06 – 2·70)	0·03
**ART Duration (years)**	1277					
≥ 10		Ref				
5–9		0·84 (0·60 – 1·16)	0·30		0·86 (0·57 – 1·25)	0·40
< 5		0·85 (0·61 – 1·20)	0·38		0·73 (0·49 – 1·10)	0·14
Unknown		1·10 (0·75 – 1·63)	0·63		1·15 (0·70 – 1·86)	0·59
**Case Contact** *	1155					
No		Ref				
Yes		0·97 (0·76 – 1·25)	0·84		1·1 (0·82 – 1·47)	0·54

* Case contact was defined as a household member treated for pulmonary tuberculosis in the past five years.

cOR: crude odds ratio; aOR: adjusted odds ratio; ART: antiretroviral therapy

# -Variables adjusted for in the model include gender, age, level of education, number of household members, tobacco smoking, secondhand smoke, house roofing, house wall, CD4 + cell count, ART duration, and case contact.

**Table 4:** Multivariable Analysis: Domestic Energy as Predictors for Tuberculosis Among 1277 HIV-Infected Outpatients Attending ART-Clinics in South Kivu, DRC. Stratification Analysis by Gender and by Estimated Years of Exposure to Biomass Fuel

Variables				Univariate		Multivariable model 1		Multivariable model 2 (by gender)				Multivariable model 3 (by years since first ugali)			
						ALL (n = 1148)		MALES (n = 282)		FEMALES (n = 963)		< 25 y (n = 329)		≥ 25 y (n = 809)	
	n	test	reference	OR	p	aOR	p	aOR	p	aOR	p	aOR	p	aOR	p
**Kitchen location**	1277	inside	outside	0·77 (0·61 – 0·99)	0·04	0·77 (0·59 – 1·01)	0·06	0·73 (0·434–1·22)	0·23	0·84 (0·61 – 1·17)	0·31	0·46 (0·26 – 0·81)	0·007	0·91 (0·66 – 1·26)	0·56
**Ventilation system**	1236	none	chimney	0·86 (0·68 – 1·08)	0·20	¨	¨	¨	¨	¨	¨	¨	¨	¨	¨
**Principal kitchen instrument**	1173	cookstove	electric/gas	1·08 (0·78 – 1·49)	0·64	0·88 (0·60 – 1·31)	0·54	0·80 (0·35 – 1·87)	0·61	0·97 (0·62 – 1·53)	0·90	1·91 (0·85 – 4·31)	0·12	0·69 (0·43 – 1·12)	0·13
**Principal kitchen instrument**	1173	three stones	electric/gas	1·38 (1·04 – 1·84)	0·02	1·08 (0·74 – 1·56)	0·70	0·89 (0·43 – 1·83)	0·75	1·07 (0·68 – 1·69)	0·77	4·05 (1·83 – 8·96)	0·001	0·66 (0·41 – 1·04)	0·08
**Only BMF for cooking**	1165	yes	no	1·26 (0·97 – 1·63)	0·07	1·66 (0·83 – 3·29)	0·15	1·62 (0·41 – 6·28)	0·48	1·62 (0·72 – 3·64)	0·24	3·24 (0·56 – 18·7)	0·19	1·62 (0·74 – 3·54)	0·23
**Only wood for cooking**	1277	yes	no	2·08 (1·14 – 3·78)	0·01	2·00 (0·70 – 5·72)	0·19	NE		1·68 (0·51 – 5·53)	0·39	3·55 (0·28–45·6)	0·33	2·00 (0·60 – 6·67)	0·26
**Improved cookstove > 10 y**	1241	no	yes	1·37 (1·08 – 1·73)	0·009	1·29 (0·93 – 1·77)	0·12	1·01 (0·53 – 1·93)	0·61	1·43 (0·98 – 2·09)	0·07	0·95 (0·48 – 1·89)	0·89	1·39 (0·94 – 2·05)	0·09
**Heating mode**	1068	wood	charcoal	1·13 (0·88 – 1·46)	0·35	¨	¨	¨	¨	¨	¨	¨	¨	¨	¨
**Lighting mode**	1275	unclean fuel	clean fuel	0·94 (0·75 – 1·18)	0·60	¨	¨	¨	¨	¨	¨	¨	¨	¨	¨
**Time with Electricity per 24h**	560	< 5h/24h	> 5h/24h	1·11 (0·77 – 1·60)	0·56	¨	¨	¨	¨	¨	¨	¨	¨	¨	¨
**Time spent in kitchen**	1227	high^§^	low	1·36 (1·07 – 1·71)	0·01	1·36 (1·06 – 1·75)	0·02	1·10 (0·67 – 1·79)	0·74	1·32 (0·97 – 1·79)	0·07	1·03 (0·60 – 1·76)	0·92	1·36 (1·01 – 1·84)	0·04
**Years since first ugali**	1277	≥ 25 y	< 25 y	1·38 (1·07 – 1·79)	0·01	1·28 (0·90 – 1·82)	0·17	1·62 (0·79 – 3·31)	0·19	0·91 (0·59 – 1·41)	0·67	¨	¨	¨	¨
**Years since first ugali**	1277	continuous (per year)		1·01 (0·99 – 1·02)	0·09	¨	¨	¨	¨	¨	¨	¨	¨	¨	¨

Total population n = 1277; lower numbers are due to missing answers. OR: odds ratio; aOR: adjusted OR; BMF: biomass fuel; NE: not estimable. All models were adjusted for age, gender (except for model 2), CD4 count, and ART duration. In addition, Model 1 also adjusted for level of education, household members, tobacco smoking, second-hand smoke, house roofing, house wall and case contact, while Model 3 also adjusted for ventilation system.

§ high = high exposure: >3h/day AND ≥ 2 times/day AND > 5 days/week.

**Table 5:** Multivariable Analysis: 24h Personal CO and Time Spent in Kitchen as Predictors for Tuberculosis Among 255 HIV-Infected Outpatients Attending ART-Clinics in South Kivu, DRC

Variables	All participants (n = 255) (96 cases / 159 controls)		Females only (n = 197) (77 cases / 120 controls)	
	Aor	p	aOR	p
**TWA CO, continuous per log10 (ppm)**	1·50 (1·01–2·23)	0·046	4.24 (1.09–16.49)	0.04
**Time spent in kitchen, high**^§^ **vs low**	2.80 (1.08–7.24)	0.03	3·33 (1·16 – 9·54)	0·03
**MAX CO, continuous per log10 (ppm)**	0·90 (0·58 – 1·32)	0·54	1.79 (0.46–7.03)	0.41
**Time spent in kitchen, high**^§^ **vs low**	2.53 (1.00–6.41)	0.05	2·76 (1·02–7·47)	0·046

CO: carbon monoxide; TWA: time-weighted average; MAX: maximum concentration; aOR: adjusted odds ratio (95% CI)

§ high = > 3h/day AND ≥ 2 times/day AND > 5 days/week

Multivariable analyses were done separately for TWA CO and MAX CO, each with adjustments for gender, reported second-hand smoke, case contact (but not for model stratifying for gender), patient on ART and CD4 + T cell count at diagnosis. Type of fuel used, and ventilation system were also tested in the analyses, but these exposure variables were not significantly associated with TB.

## Data Availability

The dataset may be made available upon reasonable request to the corresponding author.
